# HIF-Dependent *NFATC1* Activation Upregulates *ITGA5* and *PLAUR* in Intestinal Epithelium in Inflammatory Bowel Disease

**DOI:** 10.3389/fgene.2021.791640

**Published:** 2021-11-11

**Authors:** Evgeny Knyazev, Diana Maltseva, Maria Raygorodskaya, Maxim Shkurnikov

**Affiliations:** ^1^ Laboratory of Microfluidic Technologies for Biomedicine, Shemyakin-Ovchinnikov Institute of Bioorganic Chemistry of the Russian Academy of Sciences, Moscow, Russia; ^2^ Faculty of Biology and Biotechnology, National Research University Higher School of Economics (HSE), Moscow, Russia; ^3^ National Center of Medical Radiological Research, P. Hertsen Moscow Oncology Research Institute, Moscow, Russia

**Keywords:** intestinal bowel disease, hypoxia, cobalt, hydroxyquinolines, caco-2 cells, urokinase-type plasminogen activator receptor, disease markers, transcriptomics

## Abstract

Intestinal epithelial cells exist in physiological hypoxia, leading to hypoxia-inducible factor (HIF) activation and supporting barrier function and cell metabolism of the intestinal epithelium. In contrast, pathological hypoxia is a common feature of some chronic disorders, including inflammatory bowel disease (IBD). This work was aimed at studying HIF-associated changes in the intestinal epithelium in IBD. In the first step, a list of genes responding to chemical activation of hypoxia was obtained in an *in vitro* intestinal cell model with RNA sequencing. Cobalt (II) chloride and oxyquinoline treatment of both undifferentiated and differentiated Caco-2 cells activate the HIF-signaling pathway according to gene set enrichment analysis. The core gene set responding to chemical hypoxia stimulation in the intestinal model included 115 upregulated and 69 downregulated genes. Of this set, protein product was detected for 32 genes, and fold changes in proteome and RNA sequencing significantly correlate. Analysis of publicly available RNA sequencing set of the intestinal epithelial cells of patients with IBD confirmed HIF-1 signaling pathway activation in sigmoid colon of patients with ulcerative colitis and terminal ileum of patients with Crohn’s disease. Of the core gene set from the gut hypoxia model, expression activation of ITGA5 and PLAUR genes encoding integrin α5 and urokinase-type plasminogen activator receptor (uPAR) was detected in IBD specimens. The interaction of these molecules can activate cell migration and regenerative processes in the epithelium. Transcription factor analysis with the previously developed miRGTF tool revealed the possible role of HIF1A and NFATC1 in the regulation of ITGA5 and PLAUR gene expression. Detected genes can serve as markers of IBD progression and intestinal hypoxia.

## 1 Introduction

Some pathologic conditions, such as inflammatory bowel disease (IBD), including ulcerative colitis (UC) and Crohn’s disease (CD), are associated with pathological hypoxia (Taylor, 2018). UC affects only the colon, while CD affects both the small and large intestines. The exact pathogenesis of IBD is still unclear, but it is a complex pathology involving genetics, environment, microbiome, and immunome (Borg-Bartolo et al., 2020). Hypoxia leading to hypoxia-inducible factor (HIF)-signaling pathway activation is a common feature of inflammatory diseases, including IBD (Kerber et al., 2020). HIF-1*α* is protective in IBD models (Kim et al., 2021), and HIF-2*α* is essential in maintaining the immune response and regenerative capacity of the intestinal epithelium in IBD (Ramakrishnan and Shah, 2016). At the same time, HIF-2*α* but not HIF-1*α* constitutive activation leads to IBD development or exacerbation in colitis models (Xue et al., 2013; Solanki et al., 2019). HIF-1α activation in myeloid cells aggravates, and HIF-2α activation ameliorated IBD in murine models (Kim et al., 2018; Kerber et al., 2020). These results emphasize that the degree of hypoxia and HIF activation can lead to both adaptation and damage to the intestinal epithelium.

The human small intestine consists of crypts and villi, and the colon consists of crypts only. Crypts are invaginations aligned by intestinal stem cells, early progenitor transit-amplifying cells, and differentiating cells (Rangel-Huerta and Maldonado, 2017). The colon surface and small intestine villi are covered by specialized epithelium with differentiated cells, such as enterocytes and goblet cells (Singhal and Shah, 2020). The main enterocyte functions are to create a barrier between the external and internal environment and to provide regulated transport of substances between these environments (Schoultz and Keita, 2020). There is a unique oxygen gradient from the crypt to the intestinal lumen. Electron paramagnetic resonance oximetry revealed partial oxygen pressure (pO_2_) gradient from 59 mm Hg (8%) in the small intestine wall to 22 mm Hg (3%) at the villus apex and <10 mm Hg (2%) in the small intestinal lumen, whereas colon pO_2_ is 5–10 mm Hg near the crypt-lumen interface, and 11 (∼2%) and 3 mm Hg (∼0.4%) in the lumen of ascending and sigmoid colon, respectively (Singhal and Shah, 2020). Thus, intestinal cells exist under conditions ranging from mild to pronounced hypoxia, which leads to HIF stabilization and adaptation to hypoxic conditions (Pral et al., 2021). HIF stabilization in physiological hypoxia supports cell metabolism and barrier function of the intestinal epithelium (Glover et al., 2016).

Modeling of the human intestinal epithelium is possible with human colon adenocarcinoma cell line Caco-2 (Nikulin et al., 2018b). Undifferentiated Caco-2 (uCaco-2) cells actively proliferate, resembling intestinal stem cells, but contact inhibition stimulates differentiation and suppresses proliferation. Differentiated Caco-2 (dCaco-2) cells acquire enterocyte properties, such as cylindrical cell shape, formation of intercellular junctions and microvilli, specific enzyme and marker expression (Ding et al., 2021). Extracellular matrix can support cell attachment, proliferation, and differentiation (Mutsenko et al., 2017), and use of laminins specific for the intestinal basal membrane can bring the gut model properties closer to conditions *in vivo* (Nikulin et al., 2018a, 2019). Microfluidic devices with a culture medium flow that simulates blood flow create even more physiological conditions for the cell model (Samatov et al., 2016, 2017; Sakharov et al., 2019). Caco-2 intestinal model is suitable to study hypoxia effects (Maltseva et al., 2020; Nersisyan et al., 2021a). Small molecules can fit in the enzyme active center inhibiting its activity (Smirnov et al., 2011; Ivanenkov et al., 2015). Oxyquinoline derivatives can directly block the prolyl hydroxylase active center leading to HIF stabilization (Knyazev et al., 2019a; 2019b). Cobalt (II) chloride (CoCl_2_) also serves as a chemical hypoxia inductor by replacing Fe^2+^ ions in the prolyl hydroxylase active center, induction of ascorbate oxidation, and other potential mechanisms (Muñoz-Sánchez and Chánez-Cárdenas, 2019). Previously, we showed that chemical hypoxia models, induced by CoCl_2_ and OD, may serve as models of severe and mild hypoxia, respectively (Knyazev et al., 2021; Knyazev and Paul, 2021).

This work aimed to study the effects of chemical HIF activation on dCaco-2 and uCaco-2 cells as the intestinal model and compare observed changes with patterns in the intestinal epithelium of IBD patients to find hypoxic markers, relevant for IBD.

## 2 Materials and Methods

### 2.1 Cell Cultures and Treatments

Caco-2 cells were received from the Russian Vertebrate Cell Culture Collection (Institute of Cytology, Russian Academy of Sciences, St. Petersburg, Russia). The cells were incubated in Gibco minimal essential medium with L-glutamine, 20% Gibco FBS One Shot, and 1% Gibco Pen Strep (Thermo Fisher Scientific, United States ). The cells were seeded in the 6-well culture plates (TPP Techno Plastic Products AG, Germany) at a seeding density of 0.3 x 10^6^ cells per well and cultivated for 21 days to achieve cell differentiation, as previously published (Nersisyan et al., 2021a). On the last week of differentiation new portion of cells was seeded in the 6-well culture plates at the same seeding density and cultivated to 80% of confluence. The cell culture medium of differentiated and undifferentiated Caco-2 was replaced with either medium with 300 μM CoCl_2_, medium with 5 μM OD 4896–3,212 (ChemDiv Research Institute, Khimki, Russia), or fresh medium. OD stock solution was diluted in DMSO at 10 mM, so 0.5% DMSO was also added to CoCl_2_ and control medium, and cells were incubated 24 h before lysis for RNA and protein extraction.

### 2.2 RNA Extraction, Library Preparation and Sequencing

Cells were lysed in 700 μL of QIAzol Lysis Reagent (Qiagen, Hilden, Germany) with subsequent RNA extraction with miRNeasy Mini Kit (Qiagen) according to the manufacturer protocol, including on-column treatment with RNase-Free DNase Set (Qiagen). RNA was extracted in 30 μL of RNase-free water. RNA concentration was measured with NanoDrop 2000 Spectrophotometer (Thermo Fisher Scientific). A_260_/A_280_ and A_260_/A_230_ ratios were more than 2.0, and RNA Integrity Number (RIN) according to 2,100 Bioanalyzer with Agilent RNA 6000 Pico Kit (Agilent Technologies, Santa Clara, CA, United States ) was not less than 9.7 for all RNA samples.

Libraries for mRNA sequencing were prepared with TruSeq Stranded mRNA Library Prep (Illumina, San Diego, CA, United States) with 1 μg total RNA in three biological replicates for each condition. Library quality check was provided with Agilent High Sensitivity DNA Kit (Agilent Technologies). Sequencing with NextSeq 550 (Illumina) generated single-end 75-nucleotide reads. The sequencing data is available online in the Gene Expression Omnibus (GEO) with the accession numbers GSE186295 and GSE158632.

### 2.3 Sequencing Data Processing

The FASTQ file quality was assessed with FastQC v0.11.9 (Babraham Bioinformatics, Cambridge, United Kingdom). After adapter trimming with cutadapt v2.10 (Martin, 2011) reads were mapped on the reference human genome (GENCODE GRCh38.p13) with STAR v2.7.5b (Dobin et al., 2013). The count matrix was generated with GENCODE genome annotation (release 34) (Frankish et al., 2019). FASTQ files for intestinal cells in IBD and healthy control were downloaded from the online repository (E-MTAB-5464) (Howell et al., 2018). Differential expression analysis was performed with DESeq2 v1.28.1 (Love et al., 2014); false discovery rates (FDRs) were calculated by the Benjamini–Hochberg procedure. Differences were considered significant at FDR <0.05 and log fold changes modulo >1.0.

### 2.4 Gene Set Enrichment Analysis and Transcription Factors Search

Gene set processing and Venn diagram creation was made with jvenn tool (Bardou et al., 2014). Gene set enrichment analysis was performed using Fgsea package version 1.16.0 (Korotkevich et al., 2016). Gene sets were downloaded from Gene Set Enrichment Analysis (GSEA) site (http://www.gsea-msigdb.org), from The Molecular Signatures database (MSigDB 7.4) to assess the activity of hallmark gene sets (Subramanian et al., 2005; Liberzon et al., 2015). The Database for Annotation, Visualization and Integrated Discovery (DAVID) v6.8 (Huang et al., 2009) was used to reveal Kyoto Encyclopedia of Genes and Genomes (KEGG) pathways activity (Kanehisa et al., 2021).

The previously developed miRGTF-net tool was used to find transcription factors regulating genes of interest (Nersisyan et al., 2021b). The publicly available datasets of RNA sequencing data for colorectal cancer were received from The Cancer Genome Atlas Program Colon Adenocarcinoma (TCGA-COAD) cohort (Cancer Genome Atlas Network, 2012).

### 2.5 Proteome

Differentiated Caco-2 cells after 24 h incubation with CoCl_2_ and control Caco-2 cells were lysed with ice-cold lysis buffer with 4% SDS and 0.1 M DTT in 0.1 M Tris-HCl (pH 7.6) and briefly sonicated on the ice with CPX 130 Ultrasonic Processor, 130 W, 20 kHz (Cole-Parmer Instruments, Vernon Hills, IL, United States ), 30 s in pulse regimen and 30% amplitude. Total protein concentration was measured with Pierce BCA Protein Assay Kit - Reducing Agent Compatible (Thermo Fisher Scientific). Tripsinized protein samples were analyzed with the Q Exactive HF hybrid quadrupole-orbitrap mass spectrometer with nano-electrospray ionization (nESI) source operated in the positive ionization mode (Thermo Fisher Scientific), the emitter voltage of 2.1 kV, the capillary temperature of 240 C. Progenesis IQ software (Waters Corporation, Milford, MA, United States ) was used to quantify protein levels with subsequent analysis with the SearchGUI v.3.3.1 software and the HumanDB database (UniProt Release 2018_05). Differential expression was assessed with the iBAQ algorithm using MaxQuant 1.6 software (Max Planck Institute of Biochemistry, Martinsried, Germany). Differences were considered significant at a fold change >2.0 and Student’s t-test *p* < 0.05.

## 3 Results

### 3.1 General Pattern of Expression Changes in Response to Cobalt (II) Chloride and Oxyquinoline Derivative Exposure in Differentiated and Undifferentiated Caco-2 Cells

**FIGURE 1:**
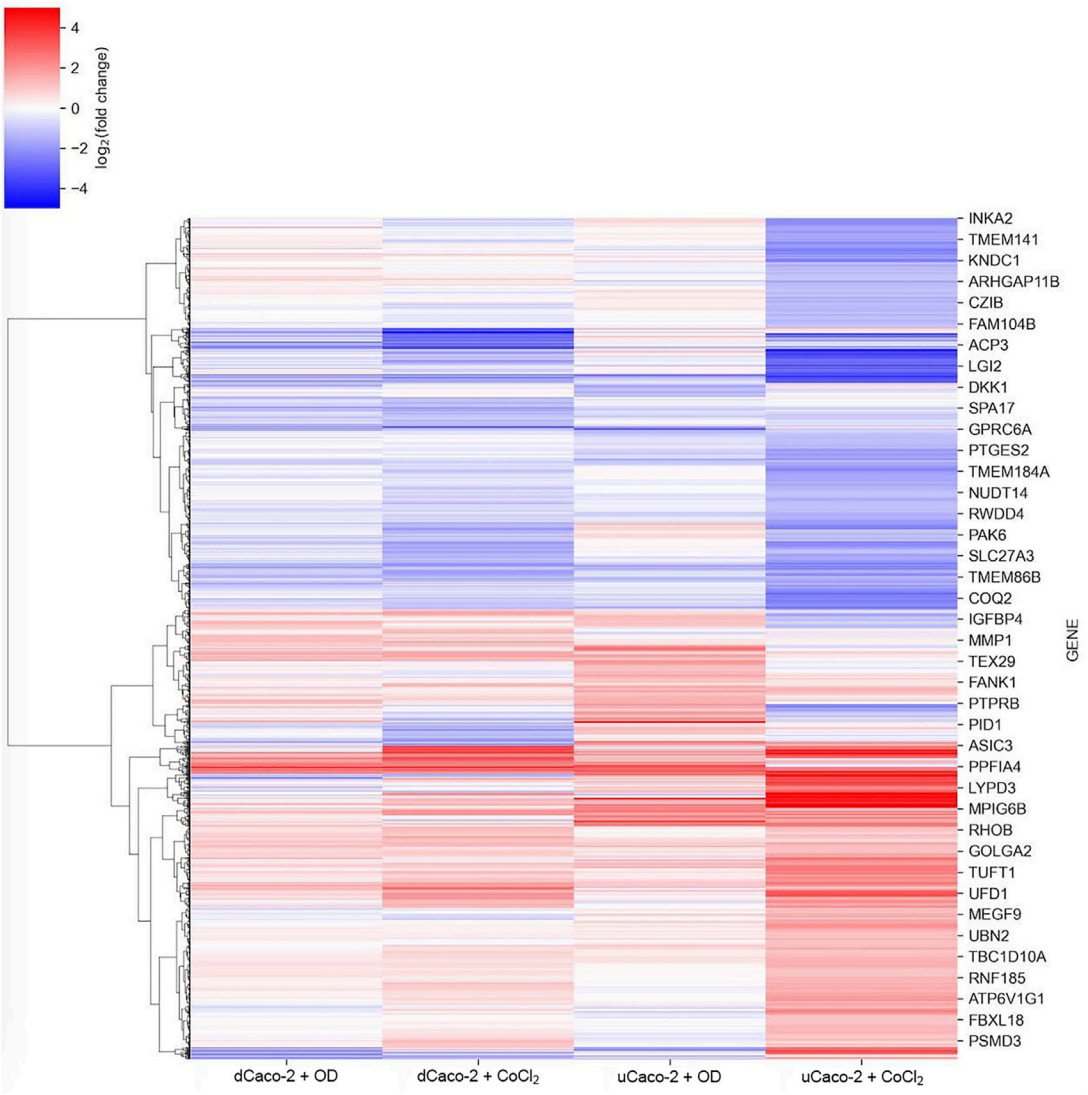
mRNA expression changes for differentiated Caco-2 (dCaco-2) and undifferentiated Caco-2 (uCaco-2) upon cobalt (II) chloride (CoCl_2_) and oxyquinoline derivative (OD) treatment. The heatmap includes genes with differential expression in at least one combination of cell line and treatment.

There is no generally accepted list of genes that should respond to HIF activation in all cells and tissues, although some studies show common patterns (Benita et al., 2009). We performed an analysis of gene expression changes in dCaco-2 and uCaco-2 upon exposure to OD and CoCl_2_ as known activators of the HIF-signaling pathway. The total number of genes whose expression changed significantly in at least one experimental group was 7,180. The pattern of expression changes was similar in all treatments with a notably stronger effect of CoCl_2_ in both cell types, the most pronounced in uCaco-2 (Figure 1 and Supplementary Table S1).

We performed gene set enrichment analysis to assess the activity of pathways and biological processes in Caco-2 cells upon HIF-pathway activation. We used hallmark gene sets from The MSigDB. Both OD and CoCl_2_ have activated HALLMARK_HYPOXIA Pathway in uCaco-2 and dCaco-2, confirming the activation of the HIF signaling pathway. Also, in all experimental settings except uCaco-2 OD stimulation, there was activation of the following pathways: HALLMARK_TNFA_SIGNALING_VIA_NFKB, HALLMARK_P53_PATHWAY, and HALLMARK_MTORC1_SIGNALING. It appears that activation of genes regulated by NF-kB in response to tumor necrosis factor *α* (TNFα) may indicate intersections between hypoxic and inflammatory effects of chemical HIF activators. CoCl_2_ and OD may activate apoptotic processes through genes involved in the p53 pathway and proliferation through genes upregulated through activation of the mTORC1 complex.

CoCl_2_ specifically activates the following pathways in both uCaco-2 and dCaco-2: HALLMARK_COMPLEMENT, HALLMARK_REACTIVE_OXYGEN_SPECIES_PATHWAY, HALLMARK_INFLAMMATORY_RESPONSE. It indicates activation of inflammatory response in Caco-2 cells, and reactive oxygen species can be generated in cells upon CoCl_2_ stimulation as a side effect apart from HIF stabilization (Muñoz-Sánchez and Chánez-Cárdenas, 2019).

### 3.2 Core Gene Set Responding to Chemical Hypoxia Simulation in Caco-2 Cells

**FIGURE 2:**
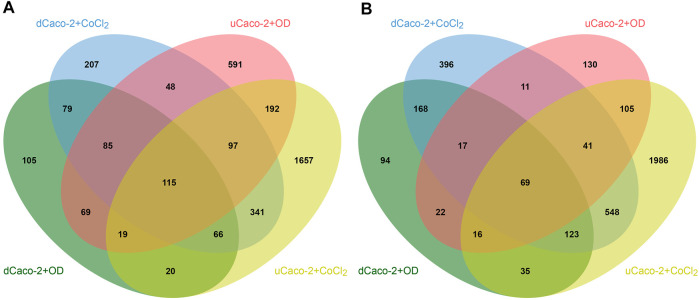
Intersections of differentially expressed genes in differentiated Caco-2 (dCaco-2) and undifferentiated Caco-2 (uCaco-2) upon cobalt (II) chloride (CoCl_2_) and oxyquinoline derivative (OD) treatment. **(A)**—upregulated genes. **(B)**—downregulated genes.

**FIGURE 3:**
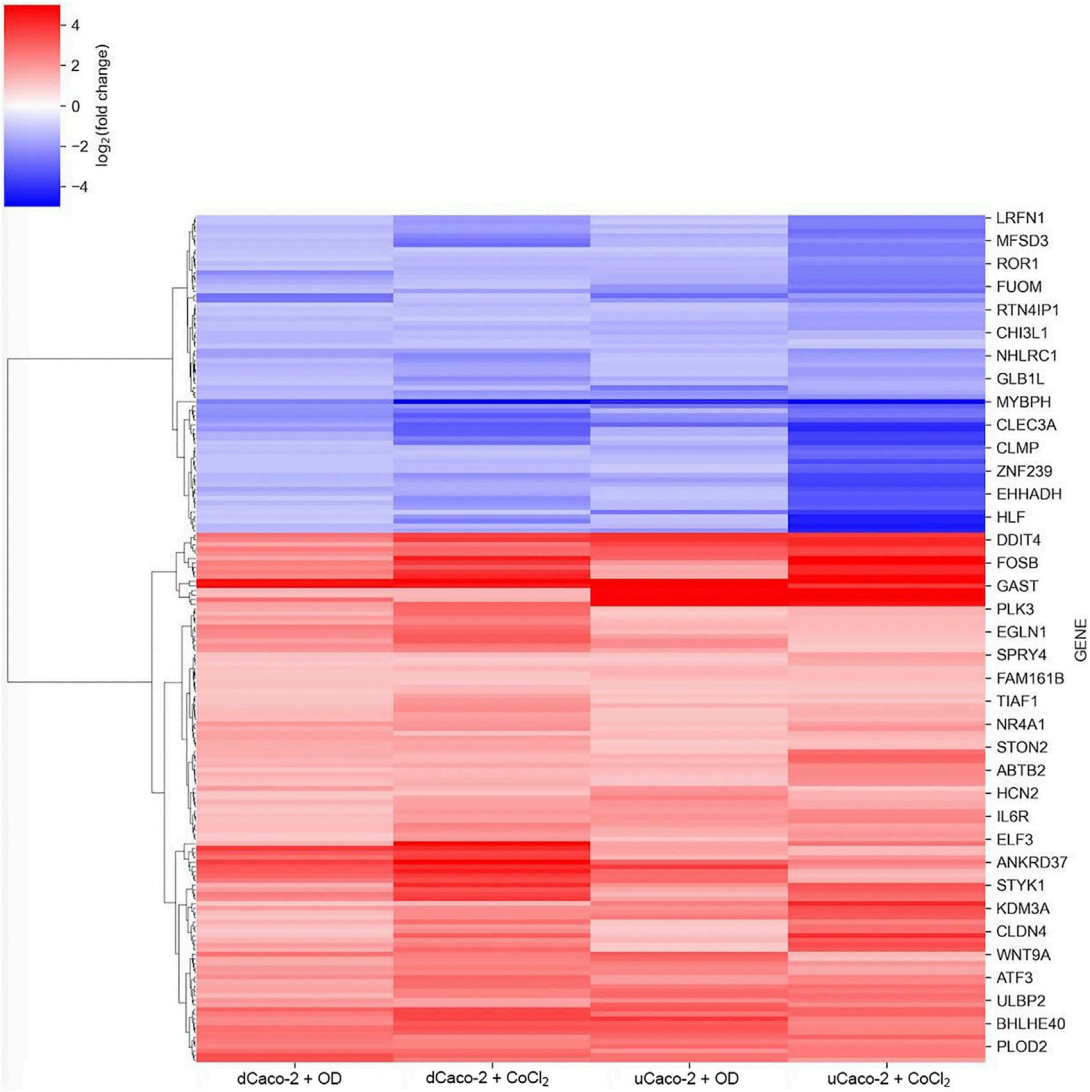
mRNA expression changes for the core gene set reacting to chemical hypoxia simulation in differentiated Caco-2 (dCaco-2) and undifferentiated Caco-2 (uCaco-2) upon cobalt (II) chloride (CoCl2) and oxyquinoline derivative (OD) treatment.

The core gene set with the same expression change direction both for uCaco-2 and dCaco-2 upon CoCl_2_ and OD treatment included 184 genes, 115 upregulated and 69 downregulated (Figure 2 and Supplementary Table S2). As for the complete list of differentially expressed genes, more pronounced expression changes were observed upon CoCl_2_ exposure, especially in uCaco-2 (Figure 3).

Enrichment Analysis of differentially expressed genes in both types of cells and both treatments using DAVID v6.8 revealed significant activation of the following KEGG pathways: Glycolysis/Gluconeogenesis (hsa00010), HIF-1 signaling pathway (hsa04066), Fructose and mannose metabolism (hsa00051), and Carbon metabolism (hsa01200), which is evidence in favor of an activation of the hypoxia signaling pathway and corresponding metabolic changes. This gene set was used as a possible HIF-associated pattern in intestinal cells exposed to hypoxia.

**FIGURE 4:**
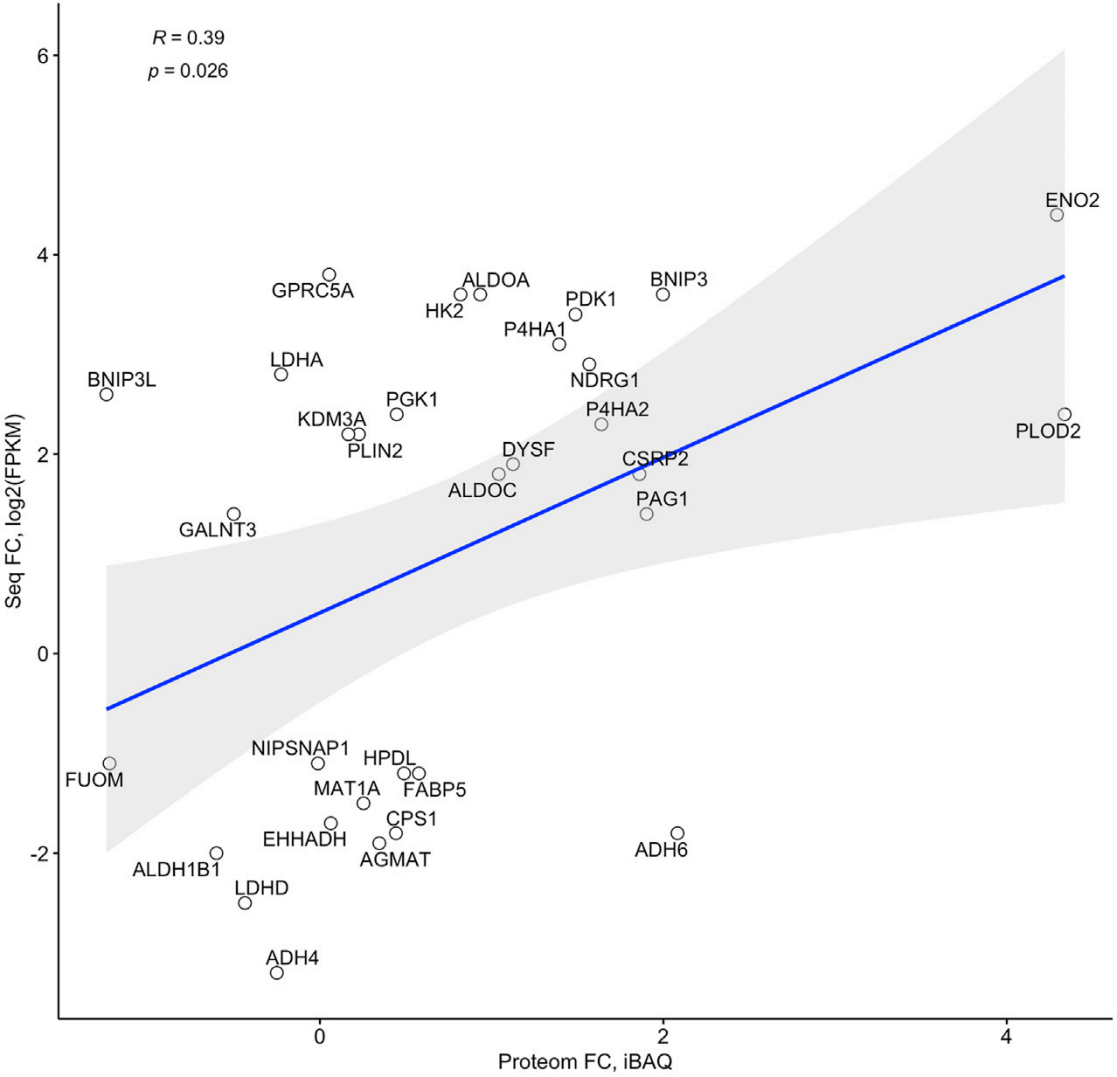
Correlation of fold changes in the RNA sequencing (Seq FC) and proteome (Proteom FC) data in differentiated Caco-2 (dCaco-2) upon cobalt (II) chloride (CoCl_2_) treatment.

Proteomic profiling of dCaco-2 upon CoCl_2_ stimulation and in control conditions revealed 3,361 proteins, of them 209 were upregulated and 28 downregulated significantly (*p* < 0.05) after CoCl_2_ stimulation. Gene set enrichment analysis for these proteins have not revealed significantly activated pathways at FDR<0.05, but *p* < 0.05 without Benjamini-Hochberg adjustment was revealed for Ribosome (hsa03010) and Endocytosis (hsa04144) KEGG pathways, suggesting an effect of HIF activation on protein synthesis and nutrient transport. Of the 184 genes on the list of HIF-associated genes, a protein product was detected for 32 genes. Analysis of fold changes in proteomic and sequencing data confirmed a significant correlation between these indicators (Figure 4). Next, we compared this gene list with specific expression changes in the intestinal epithelium of IBD patients.

### 3.3 Potential HIF-Associated Changes in Intestinal Epithelium of Patients With Inflammatory Bowel Disease

**FIGURE 5:**
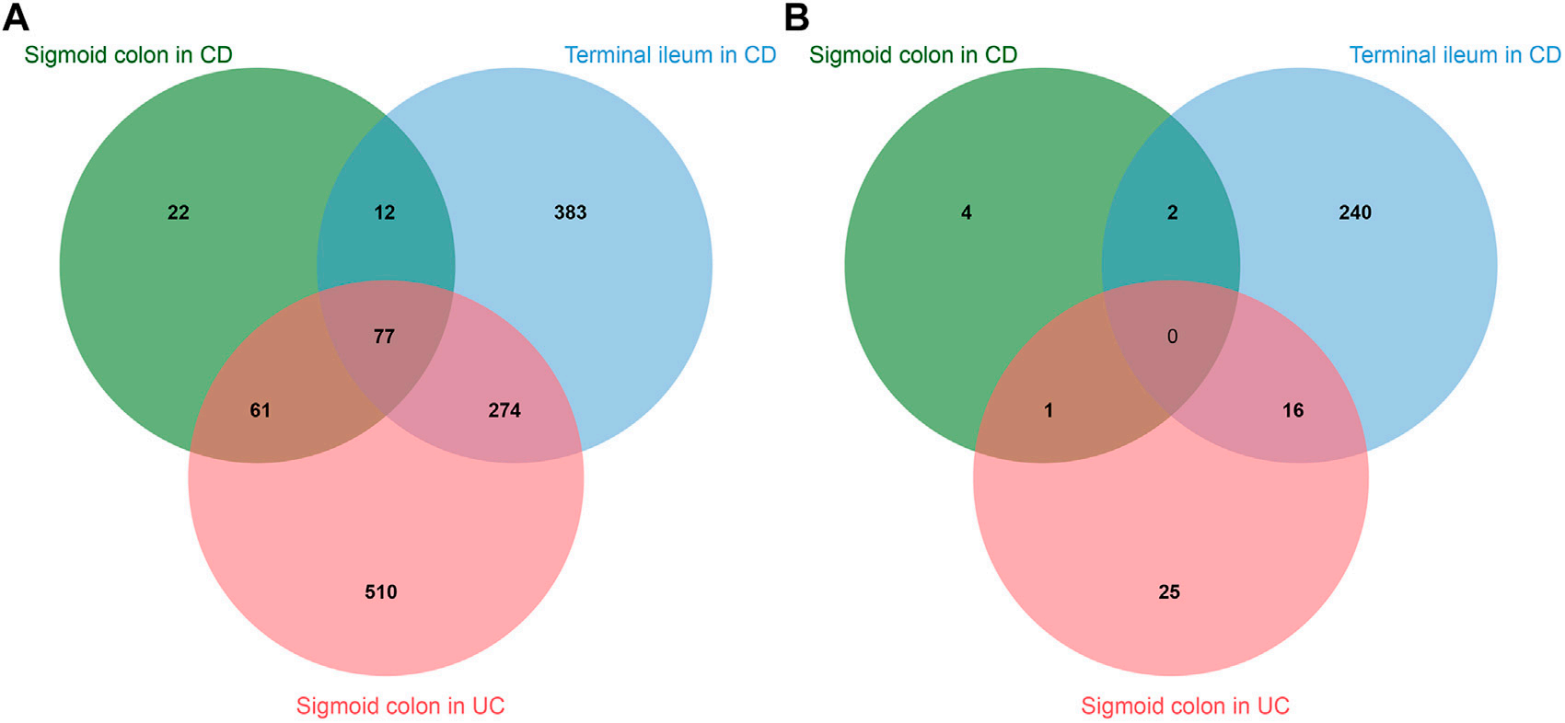
Intersections of differentially expressed genes in sigmoid colon of patients with Chron’s disease (CD), terminal ileum of patients with CD, and sigmoid colon of patients with ulcerative colitis (UC). **(A)**—upregulated genes. **(B)**—downregulated genes.

IBD includes CD, which affects both the small and large intestine, and UC, which affects the colon only. We performed an analysis of a publicly available RNA sequencing set of purified intestinal epithelial cells from pediatric biopsies including IBD and healthy controls (E-MTAB-5464) (Howell et al., 2018). We used the data on RNA expression in sigmoid colon of patients with UC and terminal ileum and sigmoid colon of patients with CD and individuals without IBD. Comparison of disease state with healthy control revealed the upregulation of 172 genes in sigmoid colon in CD, 476 genes in terminal ileum in CD, and 922 genes in sigmoid colon in UC. The downregulation was revealed for 7 genes in sigmoid colon in CD, 258 genes in terminal ileum in CD, and 42 genes in sigmoid colon in UC (Figure 5).

Gene Set Enrichment Analysis has shown activation of HALLMARK_INFLAMMATORY_RESPONSE in all groups and downregulation of HALLMARK_OXIDATIVE_PHOSPHORYLATION in sigmoid colon in UC and terminal ileum in CD, possibly indicating hypoxic response (Samanta and Semenza, 2017). The common gene set for all pathologies includes 77 genes with significant activation of the following KEGG pathways: Cytokine-cytokine receptor interaction (hsa04060) and Chemokine signaling pathway (hsa04062), which indicates the immune nature of IBD. Moreover, enrichment analysis revealed significant activation of the HIF signaling pathway in terminal ileum in CD and sigmoid colon in UC.

We analyzed the intersection of these IBD-associated genes with possible HIF-associated gene set from the experiment with chemical hypoxia models in Caco-2 cells. We noted upregulation of *ITGA5*, *MICB*, *PLAUR*, and *DYSF* and downregulation of *GSTA2*, *SLC2A2*, and *KDM8* in terminal ileum of patients with CD. Only two genes from Caco-2 experiment-derived gene set were also upregulated in sigmoid colon of patients with CD (*BHLHE40* and *PLAUR*), and eleven genes from this gene set were upregulated in sigmoid colon of patients with UC (*ITGA5, RNF183, BHLHE40, PLAUR, PFKFB3, FSCN1, SOCS3, RNF24, CSRP2, PIK3CD, DYSF*).

The only differentially expressed gene in all samples was *PLAUR*, which encodes the urokinase-type plasminogen activator receptor (uPAR) which may indicate its involvement in the course of IBD. The HIF signaling pathway according to gene set enrichment analysis was activated in terminal ileum in CD and sigmoid colon in UC, and their gene list share *ITGA5*, *PLAUR*, and *DYSF* genes.

### 3.4 Transcription Factors Regulating *ITGA5* and *PLAUR* Expression

As *ITGA5* and *PLAUR* genes are both upregulated in hypoxia model and IBD specimens and interact with each other to activate intracellular signaling (Smith and Marshall, 2010). We performed an analysis of potential transcription factors regulating both these genes with the previously developed tool miRGTF-net (Nersisyan et al., 2021b) and received 345 potential transcription regulators of *ITGA5* and *PLAUR*, including HIF1A and HIF3A. Next, we analyzed expression correlation for these transcription factors, *ITGA5*, and *PLAUR* in the mRNA-sequencing data of colorectal cancer samples from TCGA to find regulators which expression significantly correlates with both *ITGA5* and *PLAUR* in the samples of intestinal origin. *GRHL2* and *ZC3H8* have a negative correlation and *MSC*, *NFATC1*, *PRDM1*, and *SPI1* have a positive correlation with *ITGA5* and *PLAUR*. Of these transcription factors, all except SPI1 are regulated by HIF1A according to miRGTF-net. Only NFATC1 has a tendency to be upregulated in Caco-2 according to our RNA sequencing data: 1.6 times in dCaco-2 with OD and uCaco-2 with CoCl_2_, 1.9 times in dCaco-2 with CoCl_2_, and 2.9 times in uCaco-2 with OD, suggesting its possible role in *ITGA5* and *PLAUR* upregulation in hypoxia and IBD.

## 4 Discussion

There is no generally accepted list of genes that always reacting to HIF-pathway activation. Benita et al. have identified HIF-1-target genes combining genomic data from different experiments and found out core gene set of 17 genes that responded to hypoxia in all studied cell types (Benita et al., 2009). Of them, all genes responded to chemical HIF-stabilization at least in one type of cells and treatment options in our experiments, but only nine genes responded in both uCaco-2 and dCaco-2 upon CoCl_2_ and OD stimulation: *ANKRD37*, *NDRG1*, *PDK1*, *BNIP3*, *DDIT4*, *P4HA1*, *KDM3A*, *BHLHE40*, and *ALDOC*. Only nine genes from this core gene set were detected in proteomic data of dCaco-2 upon CoCl_2_ stimulation and five of them were differentially expressed on the protein level: NDRG1, PDK1, BNIP3, P4HA1, and ALDOC. Not all genes reacting to hypoxia have a HIF-binding site and some genes respond to HIF stabilization in specific cell sets (Benita et al., 2009). Hypoxia can induce gene expression not only directly through HIF, but also indirectly through regulation of microRNAs and transcription factors (Makarova et al., 2014). This makes it reasonable to search for a specific gene set responding to hypoxia in a particular cell type, as for the intestinal epithelium in our study.

CoCl_2_ and OD are known HIF inductors, acting through inhibition of HIF prolyl hydroxylases (Knyazev et al., 2021), so their effect on cells is regarded as a chemical model of hypoxia. Gene set enrichment analysis of differentially expressed genes in Caco-2 intestinal barrier model upon CoCl_2_ and OD exposure confirmed activation of hypoxia response and also an inflammatory response, proliferation, and apoptosis pathways, which are known effects of HIF activation (Corrado and Fontana, 2020).

Analysis of differentially expressed genes in the Caco-2 model of intestinal hypoxia revealed 115 upregulated and 69 downregulated genes. Enrichment analysis of this gene list confirmed HIF-1 signaling pathway activation and showed involvement of carbon metabolism and glycolysis/gluconeogenesis. HIF-dependent regulation of glycolytic, carbohydrate and fatty acid metabolism is a known process in the intestinal epithelium (Konjar et al., 2021).

Not always activation of a gene at the transcriptional level means activation at the translation level, so proteome data do not always correlate with transcriptomic data (Wang et al., 2019). We confirmed a significant correlation of gene expression at mRNA and protein level in Caco-2 cells exposed to CoCl_2_. It allows us to use RNA-sequencing data from the hypoxia experiment to search for HIF-dependent genes in samples of intestinal epithelium in IBD.

We searched for co-directed significant expression changes in the intestinal hypoxia model and intestinal epithelium in IBD. Possible HIF-associated gene list from Caco-2 model has seven genes in common with terminal ileum of patients with CD, two genes with sigmoid colon of patients with CD, and eleven genes with sigmoid colon of patients with UC. According to gene set enrichment analysis, the HIF signaling pathway was significantly activated in CD terminal ileum and UC sigmoid colon, which have three activated genes in common: *ITGA5*, *PLAUR*, and *DYSF*. *BHLHE40* was upregulated for sigmoid colon in both UC and CD. Only *PLAUR*, encoding uPAR, was activated in all three clinical specimen groups. Comparison of differentially expressed genes from the KEGG HIF-1 pathway for the Caco-2 model, CD terminal ileum and UC sigmoid colon revealed intersections, with the most prominent changes for *PFKFB3*, *TIMP1*, *ANGPT2*, *HK3*, *SERPINE1*, and *TLR4*.

*BHLHE40* encodes Basic Helix-Loop-Helix Family Member E40 that is a stress-inducible transcription factor and is activated by HIF and p53, regulating cell survival and proliferation (Kiss et al., 2020). It is a part of core response to hypoxia gene set (Benita et al., 2009) and regulates inflammatory response in the IBD model (Yu et al., 2018). *DYSF* encodes dysferlin that is a type-II transmembrane protein and is expressed in various tissues, primarily in muscle, controlling membrane repair and vesicle trafficking (Barefield et al., 2021). Its role in IBD is unclear, but elevated levels have been found in the blood of patients with IBD (Ostrowski et al., 2019).

Gene encoding uPAR was upregulated in all IBD samples. The three domains of the uPAR molecule form a cavity for the ligand of this receptor, the urokinase-type plasminogen activator (uPA). In this regard, uPAR is one of the key regulators of pericellular proteolysis, participating in the extracellular matrix remodeling and cell migration regulation (Gorrasi et al., 2020). Extracellular matrix degradation by protease systems is increased in inflamed and damaged tissues, including in IBD, which is an important component of tissue repair control (Genua et al., 2015).

uPAR can interact with vitronectin and integrins, leading to intracellular signal activation and regulating cell proliferation and survival (Smith and Marshall, 2010). An interaction between purified uPAR and *α*5*β*1 integrin has been shown *in vitro* (Wei et al., 2005), with the D3 domain of the uPAR molecule possibly forming the integrin-binding site (Chaurasia et al., 2006; Tang et al., 2008). Co-immunoprecipitation of uPAR and integrin α5β1 has also been detected (Ghiso et al., 1999; Wei et al., 2001). uPAR enhances the binding of integrin α5β1 to fibronectin (Ghiso et al., 1999; Monaghan et al., 2004) and increases cell proliferation through integrin *α*5*β*1 signaling (Aguirre-Ghiso et al., 2001; Monaghan-Benson and McKeown-Longo, 2006). The complex of *α*5*β*1 integrin and uPAR can lead to activation of the epidermal growth factor receptor signaling pathway (Aguirre Ghiso, 2002; Liu et al., 2002). There was a significant increase in the expression of the *ITGA5* gene encoding integrin α5 in Caco-2 cells upon HIF stabilization, in terminal ileum in CD, and in sigmoid colon in UC, which together with the increased expression of uPAR may lead to increased signaling through these molecules.

The role of uPAR in the IBD pathogenesis remains unclear. uPAR is expressed in intestinal crypts in normal state and IBD, although no significant expression difference has been detected (Gibson and Rosella, 1996). The intestinal nerve tissue expresses uPAR in IBD, in contrast to the nerve tissue of the healthy intestine (Laerum et al., 2008). In an animal model of IBD, increased uPAR expression at the protein and mRNA levels was detected in intestinal tissue, and this increase was predominantly due to macrophages in the intestinal wall (Genua et al., 2015). In our experiment, we observed increased *PLAUR* expression in dCaco-2 and uCaco-2 cells as a model of intestinal epithelium upon hypoxia exposure and HIF activation. Howell et al. used magnetic bead sorting for the epithelial cell adhesion molecule to sequence intestinal epithelium in IBD and healthy control (Howell et al., 2018), indicating activation of uPAR expression specifically in the intestinal epithelium.

On the one hand, HIF-activated uPAR expression stimulates extracellular matrix degradation promoting epithelial barrier destruction and cell invasion in carcinomas (Büchler et al., 2009). On the other hand, the uPAR pathway is required for efficient epithelial wound repair by stimulating extracellular matrix remodeling and cell migration (Stewart et al., 2012). It is likely that just as varying degrees of hypoxia can both promote and impair gut barrier function, so varying degrees of uPAR activation can be both damaging and regenerative. Mice with uPAR knockdown were more susceptible to the development of IBD in an experimental model (Genua et al., 2015), which may indicate a protective role of uPAR during HIF activation and inflammation. The total level of uPAR increases in patients with IBD, but the level of membrane-associated uPAR decreases as proteolysis and accumulation of the soluble form of uPAR occurs, which does not perform its functions in the activation of intracellular signaling pathways (Genua et al., 2015) This is consistent with the elevated blood level of the soluble form of uPAR in IBD (Lönnkvist et al., 2011; Kolho et al., 2012). Thus, increased uPAR expression in the intestinal epithelium may be associated not with the destruction of the extracellular matrix and damage to the intestinal wall but rather with damage adaptation, stimulation of migration and proliferation to repair the epithelial layer through interaction with integrins. Pharmacological activation of the HIF-signaling pathway stimulated intestinal epithelium repair regulating integrin expression and function (Goggins et al., 2021).

HIF-1 activation can upregulate uPAR expression (Büchler et al., 2009; Nishi et al., 2016). Hypoxia also enhances the expression of *ITGA5* (Ju et al., 2017). We identified possible transcription regulators of *ITGA5* and *PLAUR* with the previously developed tool miRGTF-net (Nersisyan et al., 2021b). HIF1A and HIF3A are both transcription factors that can regulate these genes. Analysis of correlation of all detected transcription factors with *ITGA5* and *PLAUR* in the colorectal cancer samples from TCGA revealed six genes with significant correlation. Of these six genes, only *NFATC1* expression has a tendency to be upregulated in uCaco-2 and dCaco-2 upon HIF-pathway activation, suggesting its regulatory role, and *NFATC1* expression is regulated by HIF1A according to miRGTF-net. This suggestion is supported by NFATC1 activation in hypoxic conditions (Shin et al., 2015; Xiao et al., 2020).

## 5 Conclusion

Hypoxia is a common feature in IBD pathogenesis. Chemical hypoxia induction in the Caco-2 intestinal model with cobalt (II) chloride and oxyquinoline derivative revealed potential intestinal HIF-associated gene core. Of that gene core, *ITGA5* and *PLAUR* genes were also upregulated in intestinal epithelial cells in IBD, suggesting activation of tissue regeneration program upon HIF activation. HIF-dependent upregulation of transcription factor NFATC1 is a possible mechanism of *ITGA5* and *PLAUR* activation.

## Data Availability

The datasets presented in this study can be found in online repositories. The names of the repository/repositories and accession number(s) can be found in the article/Supplementary Material.
